# Perception of mucormycosis infection among Bangladeshi healthcare workers: an exploratory cross-sectional study in the year following the COVID-19 pandemic

**DOI:** 10.1186/s42269-022-00696-1

**Published:** 2022-01-15

**Authors:** Humayun Kabir, Md. Kamrul Hasan, Mamunur Rahman, Shimpi Akter, Golam Ishraque Chowdhury, Mohammad Toyabur Rahaman Bhuya, Dipak Kumar Mitra

**Affiliations:** 1 Department of Public Health, North South University, Plot 15, Block B, Bashundhara, Dhaka, 1229 Bangladesh; 2 Department of Pharmacy, East West University, Dhaka, 1212 Bangladesh; 3 Bangladesh University of Professionals, Mirpur Cantonment, Dhaka, 1216 Bangladesh; 4 Ministry of Health and Family Welfare, Dhaka, Bangladesh; 5 Institute of Social Welfare and Research, University of Dhaka, Dhaka, 1000 Bangladesh

**Keywords:** Mucormycosis, Perception, COVID-19, Healthcare workers, Bangladesh

## Abstract

**Background:**

Mucormycosis, a severe fungal infection, is an emerging public health concern during the COVID-19 pandemic. This study aimed to investigate the perception of mucormycosis among Bangladeshi healthcare workers.

**Results:**

An exploratory cross-sectional study was carried out among the Bangladeshi healthcare workers from May 25, 2021, to June 5, 2021. The study found 422 responses from the healthcare workers of Bangladesh. Among the respondents, nearly half of them (45.26%) were doctors (*n* = 191). This study explored that the healthcare workers’ mucormycosis perception scores were significantly associated with their age, gender, profession, monthly income, marital status, job type, and death of friends and family members due to COVID-19.

**Conclusions:**

This study emphasized the healthcare workers’ mucormycosis perception along with other associated factors. The findings could help policymakers to mitigate mucormycosis and related infectious diseases emergencies in the post-COVID-19 situation.

## Background

The COVID-19, Coronavirus disease 2019, also known as SARS-CoV-2, adversely affected Bangladesh as a pandemic with around 1 million cases until July 1, 2021 (Kabir et al. 2021; Worldometer 2021). Recently, the delta variant in Bangladesh has been more transmissible (60% more) than the alpha variant, causing steeper infection rates. In the COVID-19 era, another most highlighted topic is mucormycosis, also known as black fungus, which existed to date back to 1855, emerged recently because the infection rate has elevated due to COVID-19 (Chander 2018; Hasan et al. 2021; Kamruzzaman 2021; Raut and Huy 2021). Mucormycosis, a fungal infection caused by fungi in the order Mucorales, affects people with reduced ability to fight infection and diabetes patients that help the fungi to flourish more (Greene et al. 2008; Skiada et al. 2020). According to Skaida et al., 1.7 people per million were affected by mucormycosis though the infection rate was eighty times higher in India during the COVID-19 (Skiada et al. 2020). Bangladesh detected the first COVID-19 induced mucormycosis case on May 8, 2021, and the death on May 23, 2021, whereas India reported 14,872 cases up to May 28, 2021 (Kamruzzaman 2021; Raut and Huy 2021). The Indian authorities have already stated that mucormycosis should be declared an epidemic due to the higher mortality rate (Staff 2021). However, adequate perception of healthcare workers and proper utilization consider practical measures to win in the battle of disease outbreaks (Watkins 2020). Fetansa et al., reported that the level of perception of the healthcare workers regarding any emerging diseases might be varied by different determinants (Fetansa et al. 2021).

World Health Organization (WHO) reported that around 1 out of 9 healthcare workers were affected by the COVID-19 (Linda 2020). Similarly, 11% of Bangladeshi healthcare workers were affected by 2020 (Islam et al. 2020). This year, mucormycosis has become a severe concern to the COVID-19 survived patients (Anand 2021; Kamruzzaman 2021). Therefore, mucormycosis can be a matter of concern for healthcare workers with a history of COVID-19, as they have to undergo exposure in the clinical field. On the contrary, being geographically attached to India, this became an alarming issue in Bangladesh, where the mortality rate of mucormycosis has risen to 80% in India (Staff 2021). Overuse of immunosuppressive drugs, corticosteroid therapy for the COVID-19 patients, and having comorbidities were reported to be significant factors for COVID-19 induced mucormycosis emergencies (Ahmadikia et al. 2021; Rahman et al. 2021). Also, unhealthy preventive practices, e.g., reuse of unwashed or one-time mask, could contribute to the sudden upsurge of mucormycosis infection (Sen et al. 2021).

Though mucormycosis has been described, COVID-19 associated mucormycosis is mostly rhino orbital cerebral type infection clinically manifested around the nose, eye, and even in the brain (Roden et al. 2005; Sen et al. 2021). Evidence found that immune-compromised patients at post-COVID-19 have been suffered from pulmonary mucormycosis (Alekseyev et al. 2021). However, mucormycosis is not a contagious disease in nature (Ananthaneni et al. 2013). Avoiding the risk factors and administration of antifungal agents along with better management are recognized approaches to manage mucormycosis (Skiada et al. 2018).

As front-line fighters, the Bangladeshi healthcare workers were actively involved in managing the COVID-19 situation. Similarly, they are also willing to fight against the mucormycosis infection related emergencies among COVID-19 survived patients. Thus, proper perception is considered to be the basis of excellence and wisdom in the field of outbreaks (Badran 1995). Inadequate perception of mucormycosis of healthcare workers can lead to inefficient control of the emergencies during this pandemic (Gizaw et al. 2015). In the case of COVID-19, it was found that appropriate perception contributed to managing the pandemic situation with lesser friction (Wake 2020).

Currently, no study has been found that assessed perception of mucormycosis and associated factors among Bangladeshi healthcare workers during the COVID-19 pandemic. Our study investigated the perception of mucormycosis among Bangladeshi healthcare workers with different sociodemographic, work-related, and COVID-19 related factors. The study findings may help the policymakers to respond appropriately and imply the corrective actions by improving the healthcare system of Bangladesh and the situation of the frontline healthcare workers.

## Methods

### Study design, settings, and populations

An exploratory cross-sectional study was conducted during the COVID-19 pandemic among the Bangladeshi healthcare workers between May 27, 2021, and June 3, 2021. The inclusion criteria of the respondents include; (a) the registered healthcare workers such as doctors, nurses, and others (technologist, radiologist, and medical assistant), (b) age over 18 years, (c) enrolled at clinical practices, and (d) participated by giving an online consent.

### Data collection procedure

Data were collected by a semi-structured online questionnaire (using a "Google Form) followed by convenience and snowball sampling methods. Due to the COVID-19 pandemic, face-to-face data collection was prohibited. A self-response "Google Form" questionnaire link was distributed on social media platforms or by text messages for data collection. When the participants clicked on the link, they found the study's aim and objective on the front page. The agreed respondents were transferred to the remaining part of the questionnaire. The sociodemographic and workplace-related items were presented on the second and third pages of the questionnaire, respectively. The items related to the perception of mucormycosis were delivered on the fourth page of the questionnaire. Henceforth, a total of 422 completed responses from 468 were included for the final analysis.

### Dependent and independent variable measurement

The dependent variable of this study was the perception of mucormycosis. The independent variables were sociodemographic characteristics (age, gender, residence, profession, monthly income, marital status, having children, and living status), work-related information (job type, working condition, having PPE (personal protective equipment), and direct patient contact), and COVID-19 related information (COVID-19 positive status, FNF (friend and family) members' COVID-19 positive status, and FNF COVID-19 members' death).

### Perception of mucormycosis

To examine the perception of mucormycosis, the authors adopted a six items' measure that is presented in Table 1. The items were responded to a five-point Likert scale; 1 for 'strongly disagree' to 5 for 'strongly agree.' After summing all the items, the total score was ranged from 6 to 30, whereas a higher score indicated a higher level of positive perception. For content validity, experts reviewed the questionnaire, and, based on their comments, minor modifications were performed. For construct validity, inter items correlations were checked, where the items were correlated significantly. The measures’ reliability coefficient, Cronbach α, was found 0.79, indicating an acceptable internal consistency.

**Table 1:** The items of mucormycosis perception measure

Denotes	Items
P-1	Mucormycosis is known as black fungus.
P-2	The COVID-19 increases the risk of mucormycosis infection.
P-3	Steroids treatment attributed risk of mucormycosis infection.
P-4	Immunosuppressive overuse could be a reason for mucormycosis infection.
P-5	Having comorbidities are the risk factor of mucormycosis infection.
P-6	Reusing one-time mask increases the risk of mucormycosis infection.

### Data analysis

Descriptive statistics were performed to examine the distribution of the studied variables. To find the association between studied variables and mucormycosis perception score, ‘t-test’ and ‘one-way ANOVA test’ were performed. However, before the t-test, we performed the SD test to assess the equality of variances. Multivariable hierarchical robust regression models were used to predict mucormycosis perception. The *p* value < 0.05 was considered statistically significant at a 95% confident interval. Data were analyzed by using statistical software STATA version 16. The bar charts were illustrated by Microsoft Excel version 16.

#### Results

### Descriptive statistics of studied variables

Descriptive statistics of studied variables are presented in Table 2. A total of 422 healthcare workers participated. Most of them were aged between 24 and 30 years. In terms of gender, 47.63% was male (*n* = 201). The majority of them (*n* = 372) were from urban areas (88.15%). Nearly half of them (45.26%) were doctors (*n* = 191). Maximum of the participants (54.27%) were married (*n* = 229). The participants’ having children (*n* = 106) was 25.12% and living alone (*n* = 89) was 21.09%. The majority of them (*n* = 277) did the private job (65.64%) and 31.52% worked in COVID-19 conditions (*n* = 133). Most of them (*n* = 275) had a lack of PPE (65.17%), whereas having direct patient contact (*n* = 316) was 74.88%. Almost two-thirds (*n* = 318) of them got COVID-19 infection (76%). On the other hand, 22.09% of the participants’ FNF got COVID-19 infection (*n* = 381) and died of COVID-19 infection (*n* = 93) was 22.09%.

**Table 2:** Association between studied variables and mucormycosis perception

Variables	*n* (%)	Perception		
		Mean and SD	*F*/*t* test value	*p*-value
*Sociodemographic variables*				
Age (in year)				
< 24	18 (4.27)	21.89 ± 4.90	5.34	**0.001**
24–27	168 (39.81)	24.15 ± 4.77		
28–30	146 (34.60)	24.86 ± 4.59		
> 31	90 (21.33)	25.89 ± 3.65		
Gender				
Male	201 (47.63)	24.09 ± 4.77	2.53	**0.012**
Female	221 (52.37)	25.20 ± 4.32		
Residence				
Rural	50 (11.85)	25.24 ± 4.22	0.94	0.348
Urban	372 (88.15)	24.59 ± 4.61		
Profession				
Doctor	191 (45.26)	25.88 ± 3.89	14.47	** < 0.001**
Nurse	161 (38.15)	24.0 ± 4.91		
Others	70 (16.59)	22.91 ± 4.63		
Income (monthly)				
< 20,000 BDT	89 (21.09)	22.67 ± 4.88	12.39	** < 0.001**
20000–30000 BDT	163 (38.63)	24.85 ± 4.59		
> 30000 BDT	170 (40.28)	25.55 ± 4.07		
Marital status				
Unmarried	193 (45.73)	23.92 ± 4.77	− 3.12	**0.002**
Married	229 (54.27)	25.30 ± 4.30		
Having children				
Yes	106 (25.12)	25.20 ± 4.07	− 1.48	0.140
No	316 (74.88)	24.49 ± 4.72		
Living status				
Alone	89 (21.09)	24.75 ± 4.33	0.19	0.849
With others	333 (78.91)	24.65 ± 4.64		
*Work-related variables*				
Job type				
Government	145 (34.36)	25.41 ± 4.31	2.41	**0.016**
Private	277 (65.64)	24.29 ± 4.66		
Working condition				
COVID-19	133 (31.52)	24.81 ± 5.07	0.39	0.701
Non COVID-19	289 (68.48)	24.61 ± 4.33		
Having PPE				
Yes	147 (34.83)	24.84 ± 4.49	− 1.04	0.298
No	275 (65.17)	24.35 ± 4.70		
Direct patient contact				
Yes	316 (74.88)	24.68 ± 4.68	− 0.05	0.959
No	106 (25.12)	24.65 ± 4.23		
*COVID-19 related variables*				
COVID-19 positive				
Yes	118 (27.96)	25.11 ± 4.78	− 1.23	0.218
No	304 (72.04)	24.5 ± 4.48		
FNF COVID-19 positive				
Yes	381 (75.53)	24.58 ± 4.70	0.42	0.208
No	103 (24.47)	25.00 ± 4.13		
FNF COVID-19 death				
Yes	93 (22.09)	25.51 ± 4.48	− 1.98	**0.049**
No	328 (77.91)	24.45 ± 4.57		

The bold *p*-value indicates the significant variables

FNF, friend and family

### Association between studied variables and mucormycosis perception

**Fig. 1:**
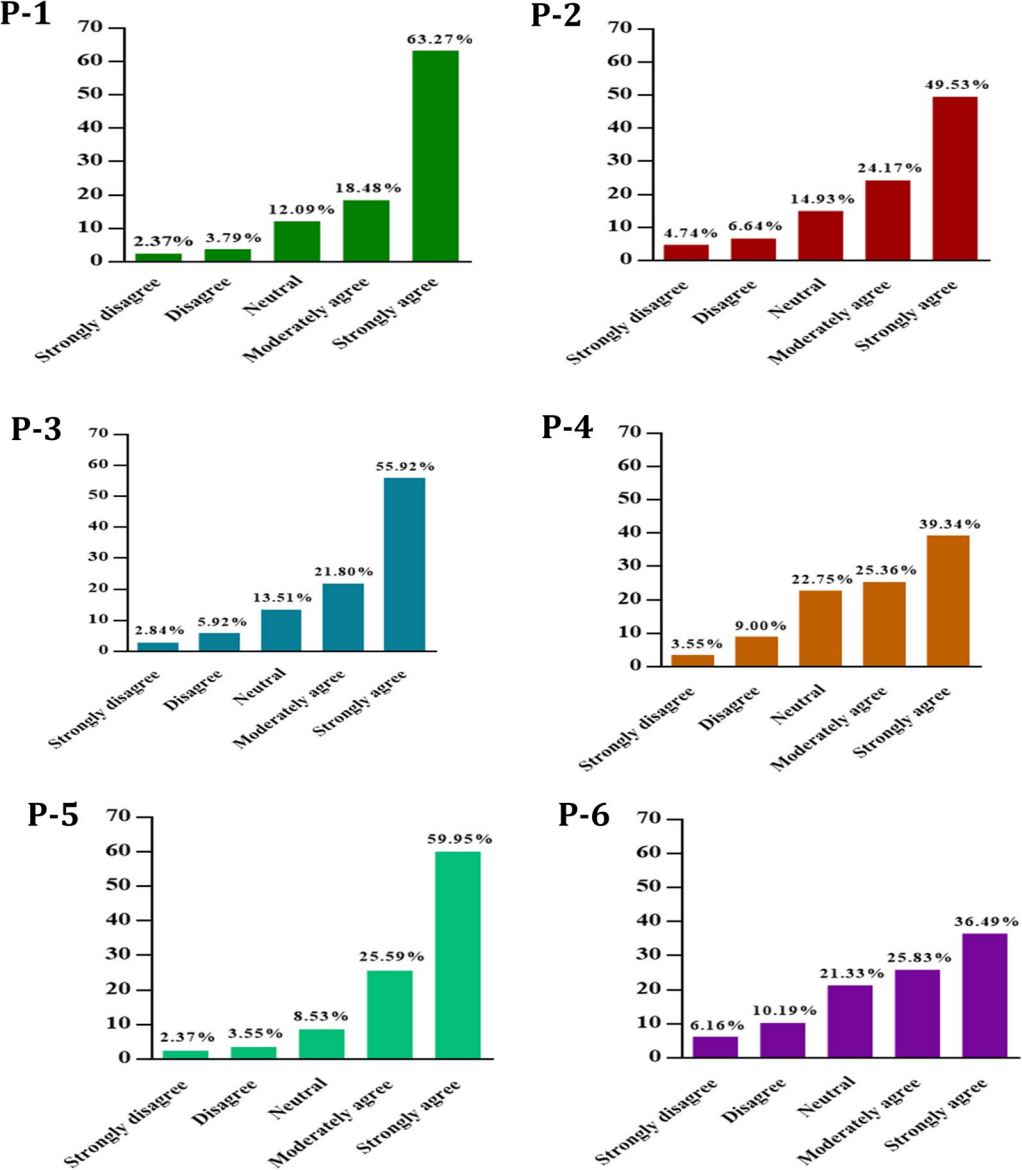
The distributions of six items’ (denoted by P-1 to P-6) mucormycosis perception measure

The association between the studied variables and the mucormycosis perception is presented in Table 2. Alongside the distribution of the responses of mucormycosis perception measure is illustrated in Fig. 1. Age was found significantly associated with mucormycosis perception (*F* = 5.34, *p* = 0.001). The mucormycosis perception score was significantly higher among the female healthcare workers than males (*t* = 2.53, *p* = 0.012). In terms of profession, this study observed a significant difference in mucormycosis perception (*F* = 14.47, *p* < 0.001). The monthly income was significantly associated with the mucormycosis perception (*F* = 14.47, *p* < 0.001). The married healthcare workers had significantly higher perception scores than unmarried (*t* = − 3.12, *p* = 0.002). The government jobholders had significantly higher perception scores than the private (*t* = 2.41, *p* = 0.016). The mucormycosis perception scores were significantly higher among the healthcare workers whose FNF died due to COVID-19 (*t* = − 1.98, *p* = 0.049).

### Predictive models of mucormycosis perception

Multivariable hierarchical robust regression models are presented in Table 3. In Block 1, the demographic variable explained an 11% variance of the perception score. Adding work-related variables in Block 2 explained 2% of the variance. After adding COVID-19 related variable in the final model (Block 3), a total explanatory variance was fixed to 14%.

**Table 3:** Multivariable hierarchical robust regression models to predict mucormycosis perception

Variables	Block 1 (β)	Block 2 (β)	Block 3 (β)
Age (in year)	0.25	0.15	0.14
Gender	− 1.18**	− 1.08*	− 1.09*
Residence	− 1.02	− 1.10	− 0.98
Profession	− 0.97**	− 1.14**	− 1.23**
Income (monthly)	0.82*	0.69	0.85*
Marital status	0.98	1.01	0.99
Having children	− 0.62	− 0.61	− 0.59
Living status	− 0.63	− 0.58	− 0.55
Tob type		− 0.72	− 0.84
Working conditions		− 0.09	− 0.14
Having PPE		0.72	0.63
Direct patient contact		− 0.18	− 0.23
COVID-19 positive			0.27
FNF COVID-19 positive			− 1.49**
FNF COVID-19 death			0.03*
*F*	6.99	5.29	4.55
*R*^2^	0.11	0.12	0.14

FNF, friend and family

***P < 0.001, **P < 0.01, *P < 0.05

## Discussion

This study found that the overall perceptions level of mucormycosis was significantly associated with the healthcare workers' age, gender, marital status, profession, monthly income, marital status, job type, and FNF death due to COVID-19. Similarly, numerous studies were conducted previously to assess the healthcare workers' perception level in different communicable diseases emergencies (Gizaw et al. 2015; Ejeh et al. 2020; Wake 2020; Dil et al. 2020; Almohammed et al. 2021; Fetansa et al. 2021; and Roupa et al. 2021).

Age was found significantly associated with mucormycosis perception. The highest mean of perception score was observed in the higher age group. Similarly, Roupa et al., found that the perception of COVID-19 was significantly higher among the older age group than the younger age group (Roupa et al. 2021). However, for healthcare workers, aging may help gaining experiences and more perception regarding any diseases (Mahmud et al. 2020).

In this study, gender was significantly associated with the perception score of mucormycosis. Female healthcare workers were perceptually more sound. A previous survey of COVID-19 indicated that the perception of female healthcare workers was more adequate than that of male healthcare workers (Almohammed et al. 2021). Moreover, Nebhinani and Saini revealed that female healthcare workers more strongly perceived NCD (non-communicable disease) related information (Nebhinani and Saini 2020).

The profession was found to be significantly associated with the perception of mucormycosis. We found that the doctors had the highest perception score compared to other healthcare workers. Similarly, Dil et al., regarding COVID-19 found that doctors had higher perception scores than other healthcare workers (Dil et al. 2020). Kanu et al., also reported that the physicians were more perceptional on the coronavirus disease than the community healthcare workers and medical technicians (Kanu et al. 2021).

This study found that married healthcare workers were more perceptional compared to unmarried healthcare workers. Studies on the coronavirus also supported our findings that married healthcare workers scored more on the coronavirus perception scale (Ejeh et al. 2020; Tsiga et al. 2021). However, Palner and Mittelmark stated that married people were mentally more vital for gaining proper health related perception (Palner and Mittelmark 2009).

The healthcare workers with government jobs were more perceptional than the private job holder healthcare workers. Similarly, Basu et al., described that the public healthcare workers were more efficient in disease perception (Basu et al. 2012). In this study, the FNF COVID-19 death was significantly associated with the perception of mucormycosis. The death of friends or family may cause anxiety in one individual life. Fitri et al., reported that social curiosity may propel acquiring more perception that helps eradicate death anxiety (Fitri et al. 2020).

In general, assessment of healthcare workers’ mucormycosis perception in terms of different determinants may strengthen the healthcare system of Bangladesh. Therefore,  to identify the factors behind the different levels of perception regarding any emerging infectious disease and taking corrective actions accordingly is essential.

### Strengths and limitations

To best of our knowledge, this is the first study that attempted to explore the mucormycosis perception among healthcare workers in Bangladesh. A better understanding of this issue can come in handy in implementing prevention measures more effectively with the emergence of mucormycosis like fungal infection.

However, there were several limitations of this study. Due to COVID-19, it was not possible to execute face-to-face interviews of participants. Thus, selection bias might be occurred owing to unintentional ruling out the healthcare workers who has no internet access. Besides, since this is a cross-sectional study, it was not possible to draw any cause-effect relationship. In addition, the usage of self-reported questionnaires might pose reporting bias.

## Conclusions

Mucormycosis is not a new infectious disease, but the emergence of mucormycosis in the COVID-19 global crisis is undoubtedly an alarming issue. In this study, the authors tried to take a snapshot of the mucormycosis perception of Bangladeshi healthcare workers. Mucormycosis perception significantly varied with respondents' age, gender, profession, monthy income, marital status, job type, COVID-19 positive, and FNF COVID-19 death. As healthcare workers are the front-line fighters worldwide in the COVID-19 pandemic, a clear perception of mucormycosis is a sine qua non.

### Recommendations

The findings could help policymakers to mitigate mucormycosis and other infectious diseases emergencies in the post-COVID-19 period and pandemic-like condition(s). This study's findings can help tailoring the preventive strategies to mitigate the health emergency in Bangladesh and generalize for other developing countries. Therefore, to overcome the health catastrophe, the policymakers can take the appropriate initiative to disseminate information about mucormycosis through training courses or online webinars with attachments to healthcare workers and emphasize the role of mass media.

## Data Availability

Dataset used in this study will be available as per request (mailing to the corresponding author).

## Abbreviations

FNF: Friend and family
COVID-19: Corona Virus-19
